# Clinical association between tacrolimus intra-patient variability and liver transplantation outcomes in patients with and without hepatocellular carcinoma

**DOI:** 10.1038/s41598-022-20636-3

**Published:** 2022-09-28

**Authors:** Hyun Jeong Kim, Juhan Lee, Jae Geun Lee, Dong Jin Joo, Myoung Soo Kim

**Affiliations:** Department of Surgery, Yonsei University College of Medicine, Seoul, Republic of Korea

**Keywords:** Hepatocellular carcinoma, Risk factors, Immunosuppression, Adverse effects

## Abstract

Tacrolimus is the mainstay of immunosuppression in liver transplantation to prevent rejection. However, the clinical use of tacrolimus is complicated by its narrow therapeutic window and significant intra-patient variability (IPV). High tacrolimus IPV is associated with overexposure and adverse effects, including malignancy. The effects of tacrolimus IPV in liver transplant recipients with and without hepatocellular carcinoma (HCC) are unknown. We investigated the association between tacrolimus IPV and transplant outcomes in 636 liver transplant patients. Tacrolimus IPV was determined by calculating the coefficient of variance (CV) of outpatient tacrolimus trough levels from 3 to 12 months after transplantation. High tacrolimus IPV was defined as CV > 30%. Patients were grouped according to tacrolimus IPV and HCC status. Among 636 liver transplant patients, 349 had HCC and 287 had no HCC. Overall survival in HCC patients was significantly reduced with high tacrolimus IPV (*P* < 0.001), whereas survival of non-HCC patients was not associated with tacrolimus IPV. Multivariable analysis confirmed the independent association between high tacrolimus IPV and overall mortality in HCC patients (HR, 3.010; 95% CI, 1.084–4.918). HCC recurred in 59 patients (16.9%) post-transplantation. After adjusting for donor/recipient factors, immunosuppression, and tumor characteristics, high tacrolimus IPV was independently associated with an increased risk of HCC recurrence (HR, 2.196; 95% CI, 1.272–3.791). High tacrolimus IPV was associated with significantly increased risks of overall mortality and HCC recurrence in liver transplant recipients with HCC.

## Introduction

Hepatocellular carcinoma (HCC) is the third most common cause of cancer-related mortality in the world and has become the major indication for liver transplantation (LT)^1–3^. Refinements in selection criteria, surgical techniques, and immunosuppressive therapies have dramatically improved short-term outcomes^4–7^. However, long-term outcomes remain suboptimal, primarily because of the adverse effects of immunosuppression, including HCC recurrence^8^. Although accumulating evidence has revealed the relationship between overexposure to immunosuppressive agents and an increased risk of HCC recurrence, optimal immunosuppressive regimens have not been clearly defined^9–12^.

Tacrolimus is a highly effective immunosuppressant and the current standard of care following LT^2,13^. Because of its narrow therapeutic window and pharmacokinetic variability, tacrolimus requires therapeutic drug monitoring (TDM)^14^. Although trough concentrations are used in most transplant centers for tacrolimus TDM, trough concentrations measured at a single time point have limited performance because of high intra-patient variability (IPV). Patients with high tacrolimus IPV may be at risk of underexposure and graft rejection or overexposure and adverse effects, including malignancy and infection^15^. Therefore, tacrolimus IPV has become recognized as a novel marker to identify solid organ transplant recipients at risk for poor outcomes^15–20^.

Despite increasing awareness of the negative influence of high tacrolimus IPV, the effects of high tacrolimus IPV in LT patients with and without HCC have not been investigated. Therefore, we conducted this study to examine the association between tacrolimus IPV and LT outcomes according to HCC status in a large cohort of liver transplant recipients.

## Results

### Baseline characteristics

A total of 636 patients who underwent LT with tacrolimus-based immunosuppression were included in this study: 349 with HCC and 287 without HCC. Baseline patient characteristics are summarized in Table 1. Compared to non-HCC recipients, HCC recipients were significantly older, were more likely to be male, were less likely to receive a liver from a deceased donor, and had a significantly lower laboratory Model for End-stage Liver Disease sodium (MELD Na) score. Hepatitis B virus (HBV) was the most common cause of the liver disease (61.3%) in the entire cohort, and the proportion of HBV was significantly higher in the HCC group than in the non-HCC group (79.1% vs. 39.7%, *P* < 0.001). The median follow-up duration was 62 months (interquartile range, 38.0–95.5 months).

**Table 1:** Baseline characteristics of patients included in the study.

Characteristics	Patients without HCC (n = 287)	Patients with HCC (n = 349)	*P*
Age, years	51.1 ± 10.0	55.4 ± 6.8	< 0.001
Female sex, n (%)	102 (35.5)	65 (18.6)	< 0.001
**Underlying liver disease, n (%)**			< 0.001
Alcoholic	98 (34.1)	26 (7.4)	
Hepatitis B	114 (39.7)	276 (79.1)	
Hepatitis C	13 (4.5)	31 (8.9)	
Biliary	18 (6.3)	0	
Others	44 (15.3)	16 (4.6)	
MELD Na score	22.8 ± 7.7	16.4 ± 6.9	< 0.001
Body mass index, kg/m^2^	24.0 ± 3.2	24.2 ± 3.1	0.396
Deceased donor, n (%)	115 (40.1)	88 (25.2)	< 0.001
Donor age, years	38.5 ± 13.8	35.2 ± 12.8	0.002
Donor female sex, n (%)	106 (36.9)	128 (36.7)	0.947
**Donor graft steatosis**			
Macrosteatosis, %	5 (0–5)	1 (0–5)	0.257
Microsteatosis, %	5 (0–5)	2 (0–5)	0.419
**Cold ischemia time, min**			
Living donor	150 (120–170)	135 (110–170)	0.11
Deceased donor	420 (345–498)	390 (326–496)	0.515
**Warm ischemia time, min**			
Living donor	57 (47–69)	60 (46–72)	0.596
Deceased donor	44 (38–50)	45 (40–55)	0.135

HCC, hepatocellular carcinoma; MELD Na, Model for End-stage Liver Disease sodium.

### Tacrolimus trough levels and intra-patient variability

A total of 6948 blood samples were analyzed for tacrolimus trough concentrations. The mean number of trough concentration measurements per patient between 3 and 12 months after LT was 11.0 ± 3.1 for HCC patients, and 10.7 ± 2.7 for non-HCC patients (*P* = 0.253). The mean tacrolimus level was 6.8 ± 1.9 ng/mL for the entire cohort, 6.7 ± 1.9 ng/mL for patients with HCC, and 6.8 ± 1.8 ng/mL for those without HCC (*P* = 0.370). The mean tacrolimus IPV was 26.7 ± 12.2% for the entire cohort, 26.8 ± 12.4% for the HCC group, and 26.5 ± 12.0% for the non-HCC group. The proportion of patients with high tacrolimus IPV was not significantly different between groups (29.9% vs. 31.0% for the HCC vs. non-HCC groups, respectively; *P* = 0.770).

To explore potential risk factors associated with high tacrolimus IPV, we performed a multivariable logistic regression analysis (Table 2). Low mean tacrolimus concentration between 3 and 12 months, serum albumin, and hematocrit at 3 months post-transplantation were significantly associated with high tacrolimus IPV.

**Table 2:** Risk factors associated with high tacrolimus intra-patient variability.

	Univariable		Multivariable	
	OR (95% CI)	*P*	OR (95% CI)	*P*
Age, per year	0.997 (0.978–1.017)	0.800		
Female sex	1.325 (0.912–1.926)	0.140	1.102 (0.722–1.684)	0.652
Body mass index, kg/m^2^	0.984 (0.932–1.038)	0.554		
MELD Na score, per point	0.997 (0.976–1.018)	0.759		
Alcoholic liver disease	1.237 (0.818–1.872)	0.314		
Hepatocellular carcinoma	0.951 (0.678–1.333)	0.770		
Mean tacrolimus concentration, ng/mL	0.820 (0.746–0.900)	< 0.001	0.825 (0.744–0.915)	< 0.001
Hematocrit, %	0.977 (0.948–1.008)	0.145	1.046 (1.003–1.091)	0.037
Albumin, mg/dL,	0.496 (0.346–0.711)	< 0.001	0.344 (0.212–0.556)	< 0.001
Total bilirubin, mg/dL	0.944 (0.793–1.124)	0.518		
Cholesterol, mg/dL	1.002 (0.999–1.006)	0.173	1.002 (0.998–1.006)	0.274
Creatinine, mg/dL	0.968 (0.694–1.350)	0.848		

CI, confidence interval; MELD Na, Model for End-stage Liver Disease sodium; OR, odds ratio.

### Tacrolimus intra-patient variability and overall survival

During the follow-up period, 101 patients (15.9%) died: 67 in the HCC group and 34 in the non-HCC group. The 1-year, 2-year, and 5-year overall survival rates were 97.4%, 92.0%, and 82.3% for the HCC group and 99.3%, 96.5%, and 90.1% for the non-HCC group (*P* = 0.020). In the HCC group, recurrent HCC (n = 38, 56.7%) and infection (n = 19, 28.4%) were the major causes of death. In the non-HCC group, the major causes of death were liver failure (n = 11, 32.4%), other malignancy (n = 8, 23.5%), and infection (n = 6, 17.6%).

**Figure 1:**
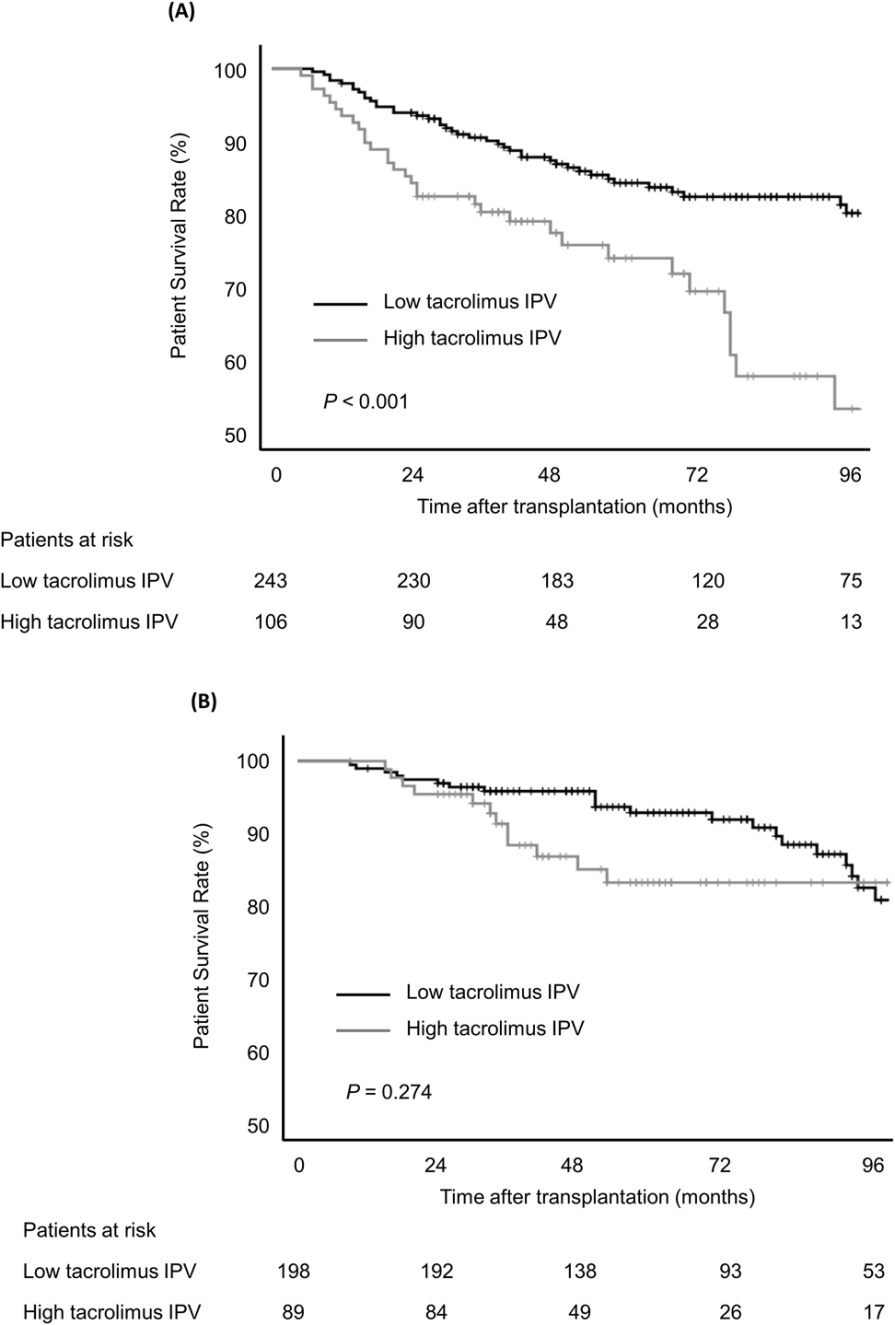
Overall patient survival stratified by tacrolimus IPV and HCC status: (**A**) patients with HCC and (**B**) without HCC.

The association between high tacrolimus IPV and patient survival was evident in the HCC group. Overall patient survival in the HCC group was significantly reduced in patients with high tacrolimus IPV (*P* < 0.001; Fig. 1A). Multivariable Cox regression analysis confirmed that high tacrolimus IPV was independently associated with higher overall mortality in the HCC group (Table 3; hazard ratio [HR], 3.010; 95% CI, 1.842–4.918; *P* < 0.001). Higher tacrolimus IPV was also associated with an increased risk of overall mortality when assessed as a continuous variable (HR, 1.049; 95% CI, 1.031–1.067; *P* < 0.001). By contrast, the overall survival of non-HCC patients was not significantly different according to tacrolimus IPV status (*P* = 0.274; Fig. 1B). In the non-HCC group, recipient age ≥ 60 years and donor age were significantly associated with an increased risk of overall patient mortality, whereas HBV-related liver disease was significantly associated with a lower risk of overall mortality.

**Table 3:** Risk factors for overall mortality in patients with and without hepatocellular carcinoma.

	Univariable		Multivariable	
	HR (95% CI)	*P*	HR (95% CI)	*P*
**Patients with HCC**				
Elderly recipient (age ≥ 60 years)	0.689 (0.382–1.242)	0.215		
Female sex	0.598 (0.286–1.251)	0.172	0.545 (0.259–1.145)	0.109
High tacrolimus IPV (CV > 30%)	2.600 (1.602–4.221)	< 0.001	3.010 (1.842–4.918)	< 0.001
Mean tacrolimus level, ng/mL	1.045 (0.919–1.188)	0.500		
Donor age, per year	1.022 (1.004–1.040)	0.014	1.024 (1.006–1.043)	0.009
Donor female sex	1.192 (0.731–1.942)	0.482		
Deceased donor	1.815 (1.113–2.959)	0.017		
MELD Na score > 25	1.503 (0.745–3.035)	0.255		
AFP > 100 IU/mL	4.296 (2.515–7.336)	< 0.001	4.833 (2.811–8.309)	< 0.001
mTOR inhibitor	0.732 (0.394–1.358)	0.323		
**Patients without HCC**				
Elderly recipient (age ≥ 60 years)	3.157 (1.516–6.575)	0.002	2.493 (1.191–5.218)	0.015
Female sex	1.303 (0.658–2.580)	0.448		
High tacrolimus IPV (CV > 30%)	1.480 (0.730–2.999)	0.277		
Mean tacrolimus level, ng/mL	1.020 (0.840–1.239)	0.842		
Donor age, per year	1.040 (1.014–1.067)	0.002	1.039 (1.012–1.066)	0.004
Donor female sex	1.076 (0.539–2.149)	0.836		
Deceased donor	2.022 (1.020–4.009)	0.044		
MELD Na score > 25	0.685 (0.334–1.406)	0.302		
HBV-related liver disease	0.281 (0.121–0.650)	0.003	0.342 (0.146–0.801)	0.014

AFP, alpha-fetoprotein; CI, confidence interval; CV, coefficient of variance; HBV, hepatitis B virus; HCC, hepatocellular carcinoma; HR, hazard ratio; IPV, intra-patient variability; MELD Na, Model for End-stage Liver Disease sodium.

### Tacrolimus intra-patient variability and biopsy proven allograft rejection

A total of 69 biopsy proven allograft rejection (BPAR) episodes occurred in 52 patients (17 [8.7%] in high tacrolimus IPV group and 35 [7.9%] in low tacrolimus IPV group). The cumulative incidence of BPAR was comparable between high and low tacrolimus IPV group (P = 0.641). The mean tacrolimus IPV was 26.9 ± 12.0% for patients with BPAR, and 26.7 ± 12.3% for those without BPAR (*P* = 0.930). The mean tacrolimus level was not significantly different (BPAR 6.8 ± 1.9 ng/mL vs. no BPAR 6.9 ± 1.5 ng/mL; *P* = 0.605).

### Tacrolimus intra-patient variability and hepatocellular carcinoma recurrence

We also analyzed the association between tacrolimus IPV and HCC recurrence after LT. As shown in Table 4, there were no significant differences in donor or recipient characteristics between the low and high tacrolimus IPV groups in patients who underwent LT for HCC. The mean tacrolimus trough level of the high tacrolimus IPV group was significantly lower than the low tacrolimus IPV group (6.1 ± 2.1 ng/mL vs. 6.9 ± 1.8 ng/mL, *P* < 0.001). Compared to low tacrolimus IPV group, high tacrolimus IPV group recipients were more likely to receive mammalian target of rapamycin (mTOR) inhibitor. Mean trough level of mTOR inhibitor was significantly higher in high tacrolimus IPV group than in low tacrolimus IPV group. Tumor characteristics, including pre-transplant alpha-fetoprotein (AFP), viable tumor number, microvascular invasion, differentiation, and maximum tumor size were comparable between the two groups.

**Table 4:** Patient and tumor characteristics according to tacrolimus intra-patient variability status in patients with hepatocellular carcinoma.

Characteristics	Low tacrolimus IPV	High tacrolimus IPV	*P*
	(CV ≤ 30%, n = 243)	(CV > 30%, n = 106)	
Age, years	55.3 ± 6.7	55.8 ± 7.2	0.518
Female sex, n (%)	46 (18.9)	19 (17.9)	0.824
**Underlying liver disease, n (%)**			0.542
Alcoholic	15 (6.2)	11 (10.4)	
Hepatitis B	196 (80.7)	80 (75.5)	
Hepatitis C	24 (9.9)	7 (6.6)	
Others	8 (3.3)	8 (7.5)	
MELD Na score	16.5 ± 6.9	16.4 ± 7.0	0.962
Body mass index, kg/m^2^	24.2 ± 3.1	24.1 ± 3.3	0.717
Deceased donor, n (%)	62 (25.5)	26 (24.5)	0.845
Donor age, y	35.2 ± 13.0	35.1 ± 12.4	0.933
Donor female sex, n (%)	94 (38.7)	34 (32.1)	0.239
**Donor graft steatosis**			
Macrosteatosis, %	1 (0–5)	5 (0–5)	0.417
Microsteatosis, %	2 (0–5)	1 (0–5)	0.434
Mean tacrolimus level, ng/mL	6.9 ± 1.8	6.1 ± 2.1	< 0.001
Tacrolimus IPV, CV %	20.4 ± 5.2	41.9 ± 11.2	< 0.001
mTOR inhibitor, n (%)	59 (24.3)	51 (48.1)	< 0.001
Mean mTOR inhibitor level, ng/mL	3.7 ± 1.4	4.3 ± 1.4	0.025
Pre-transplantation AFP, IU/mL	6.9 (3.5, 29.5)	7.6 (3.2, 25.3)	0.64
AFP > 100 IU/mL, n (%)	27 (11.1)	11 (10.5)	0.862
Microvascular invasion, n (%)	55 (22.8)	31 (29.5)	0.185
Viable tumor number	1.0 (1.0, 3.0)	2.0 (1.0, 4.0)	0.217
Maximum tumor size, cm	2.1 ± 1.7	2.3 ± 1.8	0.428
**Tumor differentiation, n (%)**			0.2
Well	27 (11.1)	10 (9.4)	
Moderate	104 (42.8)	35 (33.0)	
Poor	74 (30.5)	44 (41.5)	
Complete tumor necrosis	38 (15.6)	17 (16.0)	
Loco-regional treatment, n (%)	169 (69.5)	92 (77.4)	0.135
TACE	95	34	
RFA	14	8	
TACE + RFA	35	18	
Combined treatments	25	32	
**Number of loco-regional treatment**			0.255
1	64	28	
2	40	14	
3 or more	65	40	

AFP, alpha-fetoprotein; CV, coefficient of variance; IPV, intra-patient variability; MELD Na, Model for End-stage Liver Disease sodium; mTOR, mammalian target of rapamycin; RFA, radio frequency ablation; TACE, transarterial chemoembolization.

**Figure 2:**
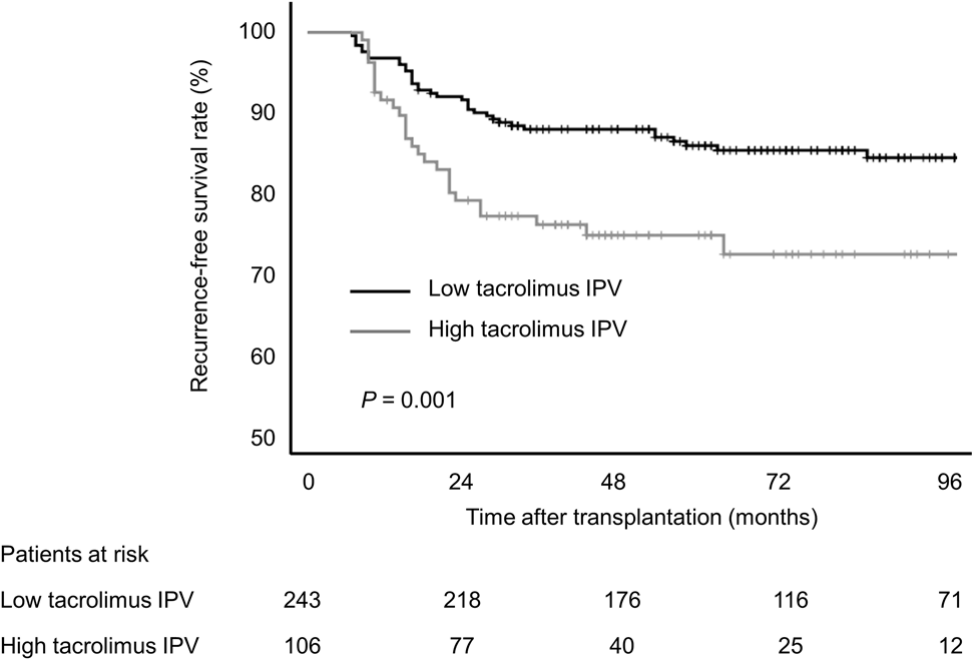
Recurrence-free survival stratified by tacrolimus intra-patient variability in patients with hepatocellular carcinoma.

HCC recurred in 59 (16.9%) patients after LT. Recurrence-free survival rates at 1, 2, and 5 years were 93.8%, 90.1%, and 86.6% for the low tacrolimus IPV group and 84.7%, 76.9%, and 74.5% for the high tacrolimus IPV group (*P* = 0.001; Fig. 2). On univariable analysis, high tacrolimus IPV was significantly associated with an increased risk of HCC recurrence. After adjusting for donor and recipient factors, immunosuppression, and tumor characteristics, high tacrolimus IPV was independently associated with an increased risk of HCC recurrence on multivariable analysis (HR, 2.196; 95% CI, 1.272–3.791; *P* = 0.005; Table 5). Higher tacrolimus IPV was also associated with an increased risk of HCC recurrence when assessed as a continuous variable (HR, 1.019; 95% CI, 1.002–1.037; *P* = 0.035). High pre-transplant AFP level (HR, 2.537; 95% CI, 1.372–4.692; *P* = 0.003), microvascular invasion (HR, 2.671; 95% CI, 1.459–4.890; *P* = 0.001), viable tumor number (HR, 1.058; 95% CI, 1.019–1.099; *P* = 0.003), and maximum tumor size (HR, 1.196; 95% CI, 1.046–1.366; *P* = 0.009) were independent risk factors for HCC recurrence, whereas recipient age ≥ 60 years was associated with a decreased risk of HCC recurrence.

**Table 5:** Risk factors for hepatocellular carcinoma recurrence.

	Univariable		Multivariable	
	HR (95% CI)	*P-*Value	HR (95% CI)	*P-*Value
Elderly recipient (age ≥ 60 years)	0.377 (0.179–0.794)	0.010	0.447 (0.210–0.950)	0.036
Female sex	0.389 (0.155–0.971)	0.043		
High tacrolimus IPV (CV > 30%)	2.280 (1.363–3.814)	0.002	2.196 (1.272–3.791)	0.005
Mean tacrolimus level, ng/mL	1.092 (0.956–1.247)	0.194		
Donor age, per year	0.997 (0.978–1.018)	0.805		
Donor female sex	1.186 (0.705–1.993)	0.521		
Deceased donor	1.057 (0.595–1.877)	0.850		
MELD Na score > 25	1.157 (0.497–2.691)	0.736		
AFP > 100 IU/mL	3.978 (2.237–7.075)	< 0.001	2.537 (1.372–4.692)	0.003
Microvascular invasion	5.741 (3.407–9.677)	< 0.001	2.671 (1.459–4.890)	0.001
Viable tumor number	1.113 (1.077–1.149)	< 0.001	1.058 (1.019–1.099)	0.003
Maximum tumor size, cm	1.338 (1.202–1.491)	< 0.001	1.196 (1.046–1.366)	0.009
Cold ischemia time, min	1.001 (0.999–1.002)	0.421		
Warm ischemia time, min	0.999 (0.984–1.014)	0.900		
mTOR inhibitor	1.167 (0.673–2.024)	0.583		
Poorly differentiated tumor	3.190 (1.901–5.355)	< 0.001	1.335 (0.754–2.366)	0.322

AFP, alpha-fetoprotein; CI, confidence interval; CV, coefficient of variance; HR, hazard ratio; IPV, intra-patient variability; MELD Na, Model for End-stage Liver Disease sodium; mTOR, mammalian target of rapamycin.

## Discussion

The rate of recurrent HCC after LT is as high as 15–20% despite careful candidate selection^21^. Although immunosuppression plays an important role in HCC recurrence, optimal immunosuppressive strategies have not been clearly defined^9–12^. In the present study, the effects of tacrolimus IPV on patient survival differed significantly between patients with and without HCC. In patients with HCC, high tacrolimus IPV was significantly associated with an increased risk of overall mortality and HCC recurrence. In contrast, tacrolimus IPV was not associated with overall survival in patients without HCC.

Tacrolimus is the mainstay of immunosuppression in solid organ transplantation to prevent rejection and graft loss^2^. However, the clinical use of tacrolimus is complicated by its narrow therapeutic window and significant IPV^22^. Since Borra et al*.* first described the negative effects of high tacrolimus IPV on graft outcomes after kidney transplantation^23^, there has been a growing body of literature supporting the association between high tacrolimus IPV and deleterious graft outcomes following non-kidney solid organ transplantation^14,19,20^.

In the LT setting, several studies have suggested a potential association between high tacrolimus IPV and increased risk of rejection, de novo donor-specific antibodies, or graft failure^17,18^. By contrast, van der Veer MAA et al*.* failed to find an association between tacrolimus IPV and immune-mediated graft injury^24^. However, previous studies used infrequent trough concentration measurements during the early postoperative period. In addition, high heterogeneity of disease severity, comorbidities, and HCC status of patients undergoing LT make it especially challenging to draw conclusions regarding the effects of tacrolimus IPV after LT^15^. To our knowledge, this is the first study to assess the effects of tacrolimus IPV on outcomes in LT recipients with and without HCC.

In this study, overall patient survival in the HCC group was significantly reduced in patients with high tacrolimus IPV. High tacrolimus IPV was also significantly associated with an increased risk of HCC recurrence. Importantly, the effects of high tacrolimus IPV on HCC recurrence remained significant in the fully adjusted model accounting for differences in tumor number, tumor size, microvascular invasion, AFP level, and mean tacrolimus trough concentration. By contrast, tacrolimus IPV was not associated with patient survival in individuals without HCC. In addition, high tacrolimus IPV was not associated with BPAR. Overall low alloimmune reactivity of liver grafts may attenuate potential adverse effects of high tacrolimus IPV, such as graft rejection and immune-mediated graft injury^24,25^.

As the immune system plays a critical role in preventing cancer development and progression^26^, use of immunosuppression may increase the risk of cancer after LT, including recurrent HCC^11,27^. In vitro and in vivo studies have demonstrated that tacrolimus enhances proto-oncogenes and cancer pathways in a dose-dependent manner^28–30^. Clinical studies have also demonstrated a dose-dependent relationship between tacrolimus and HCC recurrence^9,10^. However, optimal tacrolimus trough concentrations in LT recipients with and without HCC have not been extensively evaluated^11^. In addition, none of these studies analyzed tacrolimus IPV.

The causes of tacrolimus IPV are multifactorial and include medication non-adherence, drug-drug interactions, food intake, and gastrointestinal disorders^14,15,31^. Although medication non-adherence is a major determinant of high IPV, some degree of IPV exists regardless of adherence^22^. In this study, we observed a significant association between albumin concentration, hematocrit, and high tacrolimus IPV. This may be attributed to the documented effects of albumin concentration and hematocrit on tacrolimus distribution^32^. Regardless of the cause, high tacrolimus IPV is an important risk factor for poor outcomes in patients with HCC. Previous studies have shown that adherence-enhancing interventions can improve tacrolimus IPV^33^. Taken together, our findings suggest that HCC patients with high tacrolimus IPV require close surveillance for recurrence of HCC.

This study has several limitations worth considering. First, it is a single-center retrospective study, with the usual drawbacks of a retrospective study, as well as potentially limited generalizability. However, the single-center design has the advantage of homogeneity of immunosuppressive regimens and follow-up protocols. Second, as with any observational study, we can neither prove causality nor exclude the possibility of potential confounders. Third, information about tacrolimus adherence is lacking. Objective data regarding adherence are difficult to obtain in routine clinical practice. Nevertheless, we evaluated other potential risk factors for high tacrolimus IPV.

In conclusion, our study highlights the differential effects of tacrolimus IPV between LT recipients with and without HCC. High tacrolimus IPV significantly increased the risk of overall patient mortality and HCC recurrence after LT. These findings have important implications for managing transplant recipients, as HCC is a major indication for LT worldwide. Using tacrolimus IPV to individualize immunosuppressive treatment and employing stringent surveillance regimens for HCC recurrence may improve long-term outcomes.

## Methods

### Study population

**Figure 3:**
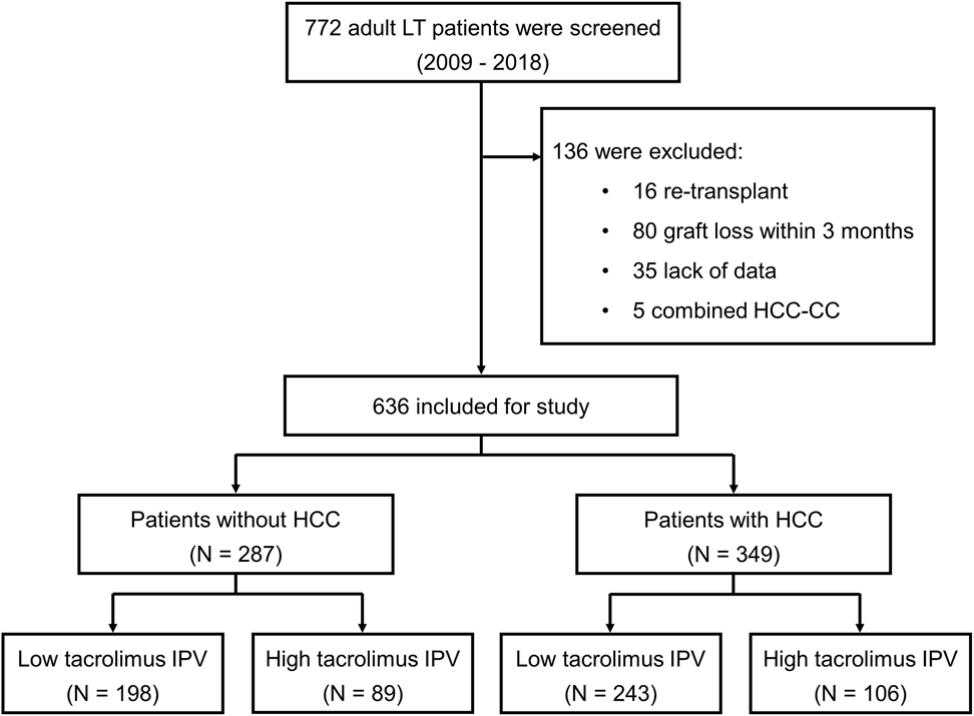
Study flow chart.

We screened 772 adults who underwent LT and received tacrolimus-based immunosuppression between January 2009 and December 2018 at the Severance Hospital, Seoul, Republic of Korea. Patients who underwent re-transplantation or who experienced graft loss within 3 months were excluded. We excluded patients with less than five tacrolimus trough concentrations between 3 and 12 months after LT or with combined hepatocellular-cholangiocarcinoma. After excluding ineligible patients, 636 transplant recipients were included in this study. These patients were categorized according to HCC status and tacrolimus IPV (Fig. 3).

### HCC selection criteria and loco-regional treatment

We accepted patients with HCC for LT irrespective of tumor number and size, in the absence of extrahepatic metastases. In patients exceeding the Milan criteria (1 lesion ≤ 5 cm or 2–3 lesions ≤ 3 cm), pretransplant loco-regional treatment was used for downstaging. Response evaluation was done at 4–6 weeks after loco-regional treatment. LT was performed if tumor number or size decreased or tumor marker decreased after loco-regional treatment. Loco-regional treatment was also used in those within the Milan criteria for bridging to LT.

### Clinical and laboratory measurements

Routine biochemical tests, including tacrolimus trough concentrations, were performed every month during the first year after LT and then every 1 to 2 months thereafter. As surveillance for HCC recurrence, serum AFP levels were measured every 1 to 2 months, and chest radiography and dynamic liver computed tomography were performed every 3 to 6 months. When HCC recurrence was suspected, magnetic resonance imaging, whole-body bone scintigraphy, or positron emission tomography was performed to establish the diagnosis.

We analyzed outpatient tacrolimus trough concentrations between 3 and 12 months. Tacrolimus trough concentrations were measured using a microparticle enzyme immunoassay: Tacrolimus II MEIA/IMx analyzer (Abbott Laboratories, Chicago, IL, USA) until May 8, 2008; Dimension RxL (Siemens, Munich, Germany) between May 9, 2008 and February 25, 2013; Architect i2000 (Abbott Laboratories) from February 26, 2013 to the end of the study. We excluded erroneously high tacrolimus concentrations (> 20 ng/mL) resulting from patients taking their morning dose of tacrolimus before blood sampling. Tacrolimus IPV was estimated by calculating the coefficient of variance (CV) using this equation: CV (%) = (standard deviation/mean tacrolimus trough concentration) × 100.

### Immunosuppression

Immunosuppression was performed according to the standard protocol at our institution^34^. Most patients received induction immunosuppression with basiliximab (20 mg on days 0 and 4 post-transplantation). Maintenance immunosuppression for all patients consisted of tacrolimus, prednisolone, and mycophenolate mofetil (MMF) or mammalian target of rapamycin (mTOR) inhibitor. The initial tacrolimus dosage (0.1 mg/kg) was administered orally. Subsequent doses were adjusted to maintain a target trough concentration between 5 and 8 ng/mL. The initial dose of methylprednisolone (500–1000 mg) was gradually reduced and replaced with oral prednisolone (5–10 mg/day) during the first 3 weeks after transplantation. MMF was initiated at 1.0–1.5 g/day, and the dose was subsequently adjusted to minimize adverse events, such as neutropenia or gastrointestinal side effects. mTOR inhibitor was usually initiated at 4 weeks after transplantation.

### Study endpoints and definitions

High tacrolimus IPV was defined as a CV > 30%^15,22,31^. HCC recurrence was defined according to radiologic evidence. Patient survival was calculated from the date of transplantation to the date of death, loss to follow-up, or December 31, 2020 (end of the follow-up period). The primary study endpoint was overall patient survival. The secondary endpoints were HCC recurrence, recurrence-free survival, and BPAR.

### Statistical analysis

Depending on the type of variable, data were expressed as frequency, mean and standard deviation, or median and interquartile range. Continuous variables were compared using Student’s t-test for parametric data or the Mann–Whitney test for nonparametric data. Chi-square or Fisher’s exact tests were used as appropriate to compare categorical variables. Multivariable logistic regression analysis was performed using high tacrolimus IPV (CV > 30%) as the outcome variable. Covariates were defined a priori and included baseline characteristics and laboratory findings at 3 months post-LT. Covariates with *P* < 0.2 in univariable analyses were entered into the multivariable logistic regression model. Overall patient survival and recurrence-free survival were analyzed using Kaplan–Meier curves and the log-rank test. Cox proportional hazard regression analyses with the backward conditional method were used to evaluate associations between tacrolimus IPV and time-to-event outcomes (overall mortality and HCC recurrence). All tests were performed two-tailed, and *P* values < 0.05 were considered statistically significant. Statistical analyses were performed using SPSS software (version 25.0; SPSS Inc., Chicago, IL, USA).

### Ethics statement

All study procedures were conducted in accordance with the Declaration of Helsinki and were approved by the Institutional Review Board of Severance Hospital (2020-2851-001). All living donations were voluntary, and all donors underwent evaluation by transplant surgeons, hepatologists, and clinical psychologists. All deceased donors were brain dead. No donor organs were obtained from executed prisoners or other institutionalized persons. Informed consent was waived by the Institutional Review Board of Severance Hospital because of the study’s retrospective design.

## Data Availability

The datasets generated during and/or analyzed during the current study are available from the corresponding author on reasonable request.
